# Detecting *Brachypodium* *distachyon* Chromosomes Bd4 and Bd5 in MH- and X-Ray-Induced Micronuclei Using mcFISH

**DOI:** 10.3390/ijms20112848

**Published:** 2019-06-11

**Authors:** Arita Kus, Joanna Szymanowska-Pułka, Jolanta Kwasniewska, Robert Hasterok

**Affiliations:** 1 Department of Plant Anatomy and Cytology, Faculty of Biology and Environmental Protection, University of Silesia in Katowice, 28 Jagiellonska Street, 40-032 Katowice, Poland; arkus@us.edu.pl; 2 Department of Biophysics and Morphogenesis of Plants, Faculty of Biology and Environmental Protection, University of Silesia in Katowice, 28 Jagiellonska Street, 40-032 Katowice, Poland; joanna.szymanowska-pulka@us.edu.pl

**Keywords:** BAC clones, *Brachypodium distachyon*, chromosome painting, mcFISH, micronuclei, model grass, molecular cytogenetics, mutagens

## Abstract

Micronuclei are biomarkers of genotoxic effects and chromosomal instability. They are formed when chromosome fragments or whole chromosomes fail to disjoin into daughter nuclei. We present qualitative and quantitative analyses of the involvement of specific chromosome regions of chromosomes Bd4 and Bd5 in the formation of micronuclei of *Brachypodium distachyon* root tip cells following maleic hydrazide (MH) treatment and X-radiation. This is visualised by cytomolecular approaches using bacterial artificial chromosome (BAC)-based multicolour fluorescence in situ hybridisation (mcFISH) in combination with 5S and 25S rDNA probes. The results showed that the long arm of submetacentric chromosome Bd4 forms micronuclei at twice the frequency of its short arm, suggesting that the former is more prone to double-strand breaks (DSBs). In contrast, no difference was observed in the frequency of micronuclei derived from the long and short arms of submetacentric chromosome Bd5. Interestingly, the proximal region of the short arm of Bd5 is more prone to DSBs than its distal part. This demonstrates that 5S rDNA and 35S rDNA loci are not “hot spots” for DNA breaks after the application of these mutagens.

## 1. Introduction

The integrity and stability of DNA in a nucleus are essential for the correct execution of cellular metabolism at interphase and the accurate distribution of genetic information during cell division. Interruptions in the continuity of DNA strands, which result in structural chromosomal aberrations, can be caused by different genotoxic agents. Chromosomal aberrations, including abnormalities in the structure and/or number of chromosomes, are considered to be hallmarks of genome instability. Despite the fact that recent studies have provided some insights into the effects of various mutagens in plant cells [1,2,3,4], our knowledge of potential double-strand break (DSB) “hot spots” in the plant genome is still limited. Hsu [5] proposed a bodyguard hypothesis, which states that euchromatin centrally localised in the nucleus may be protected from chemical agents and X-radiation by surrounding constitutive heterochromatin at the nuclear periphery. Although this hypothesis gains support from studies of ethyl methanesulfonate (EMS)-treated maize cells, it does not appear to apply to *Crepis capillaris* cells subjected to maleic hydrazide (MH) treatment [6]. Moreover, it has been demonstrated that heterochromatic regions comprising highly repetitive DNA sequences may harbour breakage “hot spots” in plants [7,8]. In accordance with the “bodyguard” hypothesis, it has been shown that *Drosophila* mutants with higher proportions of heterochromatin display less DNA damage, a higher viability after irradiation [9], and a longer lifespan [10] compared to a wild type. Analysis of gamma-ray treated human fibroblasts also reveals a protective role of condensed chromatin against DNA damage [11]. Other factors could be responsible for the non-random distributions of chromosome aberrations, such as the spatial organisation of the nucleus at interphase, the diverse transcriptional activity of specific chromosome regions, and chromosome size [12].

Different assays can be used to gauge mutagenicity, such as screening for chromosome aberrations during cell division. However, an analysis of individual dividing cells is very arduous and time consuming [13], and most mutagens decrease mitotic activity [14,15]. As an alternative to screening metaphase chromosomes, a micronucleus assay was developed as a fast but reliable method for measuring the damage that is caused by genotoxic agents [16]. Micronuclei are small, extranuclear bodies that are formed from chromatin chunks arising from chromosome breakage or the loss of entire chromosomes that failed to attach to the mitotic spindle at metaphase [17]. Chromosome mis-segregation can also be caused by kinetochore damage, failure of the cell cycle control system, or centromeric DNA hypomethylation [18]. Some micronuclei might also be derived from breakage of anaphase bridges formed from dicentric chromosomes, concatenated ring chromosomes, the union of sister chromatids, unresolved sister chromatid connections, or chromosomes that had been joined by telomere fusion [19].

Although the micronucleus assay was first used by Evans et al. in 1959 [20] in gamma-ray treated root tips of kidney beans, the identity and origin of micronuclei are still unclear. The identification of micronuclei in plants is limited due to the dearth of fluorescence in situ hybridisation (FISH) probes that can target specific chromosomes and their regions. As a consequence, less specific repetitive DNA, such as centromeric, telomeric, and rDNA sequences are more commonly used as FISH probes to determine the origin of micronuclei. These DNA probes have proven to be useful in determining whether entire chromosomes or just their fragment(s) are involved in the formation of micronuclei in species such as barley [21,22,23], *C*. *capillaris* [24], and *Brachypodium distachyon* (Brachypodium) [25].

Chromosome painting (CP) is an advanced variant of FISH that is able to detect entire chromosomes or specific chromosome regions. CP has shown that all human chromosomes can form micronuclei [23,26], but more importantly that particular chromosomes are more likely to form micronuclei [27,28,29,30,31], which could be connected with chromosome size and gene density [32], as well as with chromatin organisation [33,34]. Other chromosomes can be involved in micronuclei formation but through an apparently random process [27,35]. CP may be difficult in plants due to the presence of large amounts of repetitive DNA on all chromosomes. To our knowledge, CP has only been used successfully once in mutagenesis studies of plants cells, namely for the analysis of maleic hydrazide (MH)- and X-ray-induced micronuclei of Brachypodium [25].

Brachypodium has many useful attributes, such as a small (~300 Mb) and sequenced nuclear genome, a small amount of repetitive DNA, a low chromosome number, small stature, short life cycle, and self-fertility, which makes it a suitable model organism for cereals and other grasses [36,37,38]. Previously, we analysed qualitatively and quantitatively the involvement of specific chromosome segments in micronuclei following MH treatment and X-radiation, in order to infer potential “hot spots” of mutagen-induced DNA breaks in Brachypodium. Using multicolour FISH (mcFISH) with repetitive (5S rDNA, 25S rDNA, telomeric, and centromeric) [39] and low-repeat bacterial artificial chromosome (BAC) clones specific for the Bd1 chromosome [25], we observed that the majority of micronuclei comprised large, acentric fragments showing that DSBs rarely occur in distal chromosome regions. By selection of the longest chromosome its metacentric shape allowed us to conclude that DSBs are not randomly distributed along its two chromosome arms. In order to determine if the same rule applies to other chromosomes of the complement, we here selected the submetacentrics Bd4 and Bd5. We show the composition and putative origin of micronuclei from the long and short arms of these two chromosomes in Brachypodium root-tip meristematic cells after MH treatment and X-radiation, and use these observations to infer the distribution of DSBs.

## 2. Results and Discussion

### 2.1. DSBs from MH Treatment and X-Radiation Are Non-Randomly Distributed along the Arms of Chromosome Bd4

**Figure 1:**
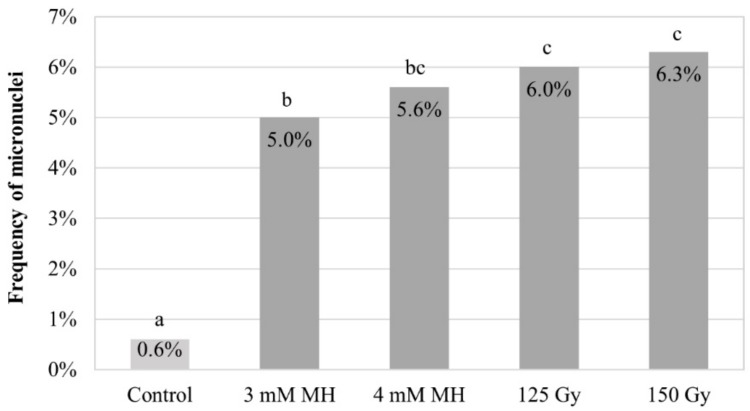
Frequencies of micronuclei in *Brachypodium distachyon* (Brachypodium) root tip meristematic cells after maleic hydrazide (MH) treatment at two concentrations and X-radiation at two doses. Means followed by the same letter (a, b, or c) are not significantly different from each other based on the parametric analysis of variance and post hoc LSD test (*p* < 0.05).

Analysis of the total frequencies of the micronuclei in control and treated Brachypodium root meristematic cells was derived following DAPI (4′,6-diamidino-2-phenylindole) staining. The results showed the clastogenic effects of MH and X-radiation in comparison to the control (Figure 1). The frequencies of micronuclei depended on the mutagen type and concentration. These varied from 5.0% for 3 mM MH to 5.6% for 4 mM MH, and from 6.0% to 6.3% for 125 Gy and 150 Gy of X-radiation, respectively. These results were in agreement with our previous findings [25,39] and demonstrate the sensitivity of Brachypodium cells to mutagens.

**Figure 2:**
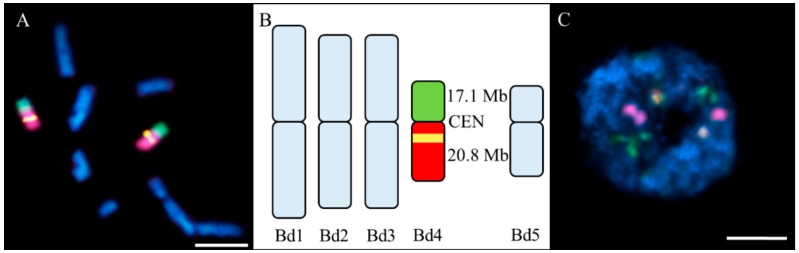
Results of fluorescence in situ hybridisation (FISH) with the bacterial artificial chromosome (BAC) clones labelling the short (green) and long (red) arms of the Bd4 and a 5S rDNA probe (yellow) in the Brachypodium control material. The chromatin was stained with DAPI (4′,6-diamidino-2-phenylindole) (blue). (**A**) Painted mitotic metaphase chromosomes; (**B**) ideogram of the chromosomes derived from (**A**) with total lengths of the BAC pools (CEN, centromere); and (**C**) exemplary interphase nucleus with no micronuclei. Scale bar = 5 µm.

We used low-repeat BAC-based probes to paint the entire arms of the Bd4 chromosome pair, together with a 5S rDNA probe. Discrimination between the two Bd4 arms was achieved by differential labelling with arm-specific BAC contigs (Table S1). This chromosome is submetacentric with a long (L) to short (S) arm length ratio (L/S AR) of 1.8, and the single 5S rDNA locus occupies the proximal part of the long arm [40]. Chromosome preparations from untreated material were used as a control to verify the specificity of the probes (Figure 2). Both the short (green) and long (red) arm-specific BAC probes gave a continuous painting signal, except for the 5S rDNA sequence (yellow), which was excluded from the long arm-specific BAC pool and was visualised separately with a specific probe (Figure 2A). In the interphase nuclei, they occupied specific and well-defined territories with clear and distinct signals with the 5S rDNA probe (Figure 2C).

**Figure 3:**
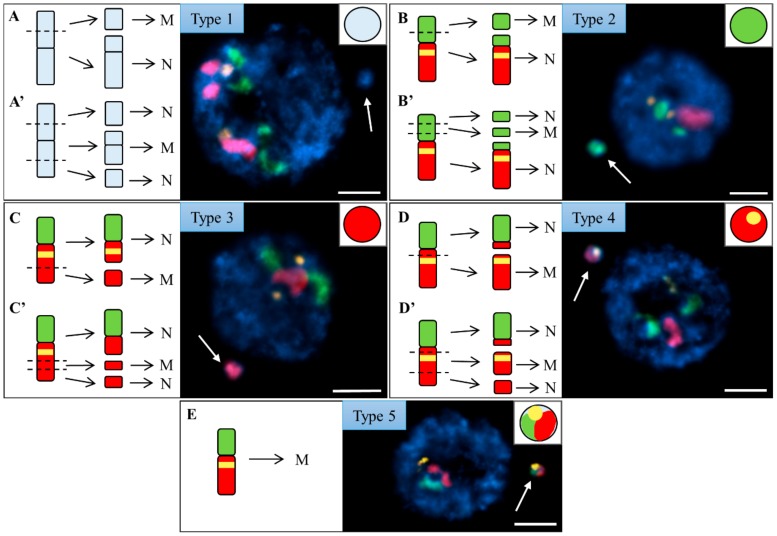
Brachypodium interphase nuclei with micronuclei induced by MH treatment and X-radiation, mcFISHed with the short (green) and long arm (red) Bd4-specific BAC probes and a 5S rDNA probe (yellow). The chromatin was stained with DAPI (blue), and white arrows indicate the micronuclei. Various types of micronuclei are distinguished and their detailed composition is described in the main text. The diagrams next to the photomicrographs show the putative origins of the micronuclei from (**A**–**D**) distal or (**A’**–**D’**) interstitial chromosome regions or from (**E**) the entire, laggard chromosome Bd4. N, nuclei. MN, micronuclei. Transverse dashed lines indicate the double-strand breaks (DSBs). Scale bar = 5 µm.

Micronuclei were classified into five types based on the presence or absence of specific FISH signal(s) (Figure 3). A type 1 micronucleus has no FISH signals and was derived from the distal (Figure 3A) or interstitial (Figure 3A’) fragment(s) of chromosomes Bd1, Bd2, Bd3, or Bd5. The presence of one signal derived from the BAC pool spanning the short arm of Bd4 in a type 2 micronucleus suggested that either one (Figure 3B) or two (Figure 3B’) DSBs occurred within the region of the chromosome covered by this BAC pool. By contrast, a type 3 micronucleus had one signal relating to the long arm Bd4-specific BAC pool. This indicates that it was formed from the distal (Figure 3C) or interstitial (Figure 3C’) fragment of the long arm of Bd4. A type 4 micronucleus was characterised by the presence of one 5S rDNA signal and one signal derived from the long arm-painting Bd4 probe (red). Such a composition showed that it was formed from a large, distal (Figure 3D) or interstitial (Figure 3D’) fragment of the long arm of Bd4. Micronucleus type 5 was the most structurally complex, and consisted of one signal of 5S rDNA and two signals from different BAC pools. Such a composition suggested that it arose from one intact laggard chromosome Bd4 (Figure 3E).

**Figure 4:**
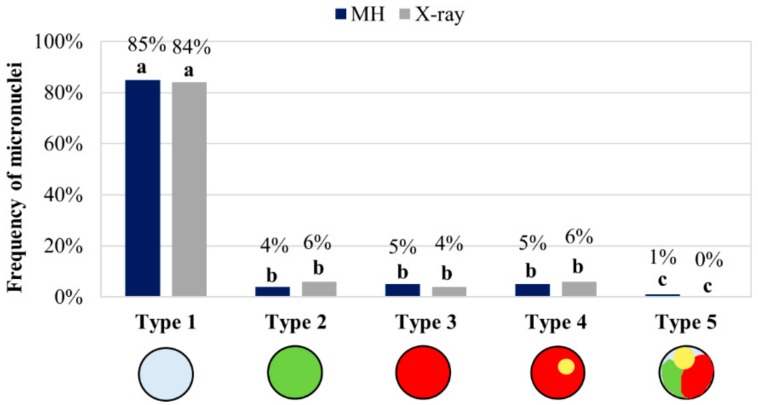
Frequencies of the micronuclei with the signals of the BAC pools painting the entire arms of Bd4 and of the 5S rDNA probe in Brachypodium cells after MH and X-ray treatments. The datasets for two concentrations of MH and two doses of X-radiation were combined. Means followed by the same letter (a, b, and c) are not significantly different from each other based on the parametric analysis of variance and post hoc LSD test (*p* < 0.05).

The relative frequencies of the five types of micronuclei resulting from MH and X-ray treatments are shown in Figure 4. Data from the two different concentrations of MH and two different doses of X-rays were each combined as they were indistinguishable.

Statistical analyses using ANOVA and LSD multiple comparison tests revealed significant differences (*p* < 0.05) in the frequency of various micronuclei types. After MH treatment and X-radiation, the frequencies of micronuclei type 1 were significantly higher than the frequencies of all of the other types (Table S3). Although this difference was visually obvious on the diagram (Figure 4), it was also statistically validated. Moreover, the frequencies of micronuclei type 5 where both mutagens had been applied were significantly different from all of the other types. No statistically significant differences in the frequencies were detected for the other micronuclei (types 2, 3, and 4).

A quantitative analysis of all five of the types of micronuclei described above revealed that FISH-negative micronuclei were the most frequent and contributed to 85% of all of the micronuclei after the MH and 84% after X-ray treatments (Figure 4). This is not unexpected given that 8 of the 10 (80%) chromosomes of the complement are unlabelled. The use of Bd4 chromosome arm-specific probes enabled the precise identification of the individual fragments of Bd4 in the micronuclei, and also the involvement of the short and long arms of this submetacentric chromosome. Interestingly, although the two arms were of different lengths, the frequencies of micronuclei type 2, (which had a BAC signal specific for the short arm of Bd4) and micronuclei type 3 or 4 (which had long-arm-specific signals), were similar. The total frequencies of micronuclei types 3 and 4 demonstrated that the long arm was involved in micronuclei formation at relatively high frequencies (10%) after both MH treatment and X-radiation. The short arm of Bd4 formed micronuclei at about a two-fold lower frequency after the MH treatment (4%) and X-radiation (6%) than the long arm. These results correlated with the morphometric analyses of this chromosome showing that the long arm of Bd4 is 1.8 times longer than its short arm [40]. Thus, the long arm could be more prone to breakage because it presents more potential targets for the mutagens. These observations also suggested that the involvement of specific chromosome segments along the submetacentric chromosome Bd4 that was crucial for inducing DSBs in Brachypodium after mutagens had been applied. These data aligned with previous reports from our group [25], which showed no significant difference in the involvement of the top and bottom arm of the almost metacentric chromosome Bd1 in micronuclei formation after X-ray and MH treatments.

The use of 5S rDNA as a probe showed how often the large or small fragments of the long arm of Bd4 constitute micronuclei. Micronuclei type 3 (5%, 4%) and 4 (5%, 6%) was present at almost the same frequency after MH treatment and X-radiation, respectively. This suggests that DSBs occur at a similar frequency in both the distal and proximal regions of Bd4, and that the 5S rDNA sequences are not preferentially involved in micronuclei formation. The composition of micronuclei was analysed previously in barley cells that had been subjected to MH treatment, and most were formed from large, acentric fragments that included the rDNA loci located in the interstitial or pericentric parts of the chromosomes [22]. We observed no micronuclei with only 5S rDNA signals after MH treatment or X-radiation, implying that DSBs do not occur preferentially within this chromosome region.

Type 5 micronuclei had the lowest frequency (1%) after MH treatment, and were not detected after X-radiation. This implies that the entire, laggard chromosome Bd4 is rarely involved in micronuclei formation. MH is a clastogenic agent and causes mitotic spindle defects, whereas the impact of X-rays on the mitotic apparatus is not well known. Our results confirm the destructive action of MH on the mitotic spindle, which resulted in chromosome Bd4 being excluded from the daughter nucleus at the completion of telophase during mitosis. The frequencies of the five types of micronuclei did not depend upon the chemical and physical mutagens used, despite their different mode of action. These results are consistent with a similar study of chromosome Bd1, which showed that the relative frequencies of the different types of X-ray-induced micronuclei are no different to those that are induced by MH [25,39].

### 2.2. The Proximal Region of the Short Arm of Bd5 Is More Prone to DSBs Than Its Distal Part

**Figure 5:**
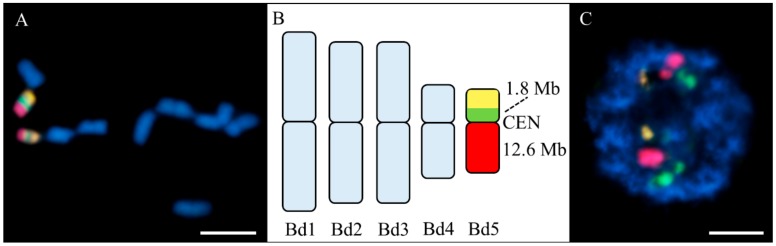
FISH with BAC clones labelling the short (green) and long (red) arms of Bd5 and a 25S rDNA probe (yellow) in the control material. The chromatin was stained with DAPI (blue). (**A**) Mitotic metaphase chromosomes; (**B**) ideogram of the chromosomes from (**A**) with total lengths of the BAC pools (CEN, centromere); and (**C**) exemplary interphase nucleus with no micronuclei. Scale bar = 5 µm.

In order to further explore any site-specificity of DSBs in the short and long arms of the submetacentric chromosomes of the Brachypodium complement, we decided to obtain the relative frequencies of micronuclei types derived from the whole chromosome or chromosome fragment(s) of Bd5. This chromosome is submetacentric with an L/S AR that is equal to 2.25 [40]. To paint it along its entire length, pools of BAC DNA were combined into arm-specific probes (Table S2). Both short (green) and long (red) arm-specific Bd5 probes produced continuous linear signals along mitotic metaphase chromosome preparations (Figure 5). Additionally, a 35S rDNA (yellow) sequence was used as a probe, which spanned the entire satellite from the distal part of the Bd5 short arm to the secondary constriction. When hybridised to interphase nuclei, short and long arm Bd5-specific BAC pools are visualized at a higher mapping resolution, and are accompanied by two signals of the 25S rDNA probe.

**Figure 6:**
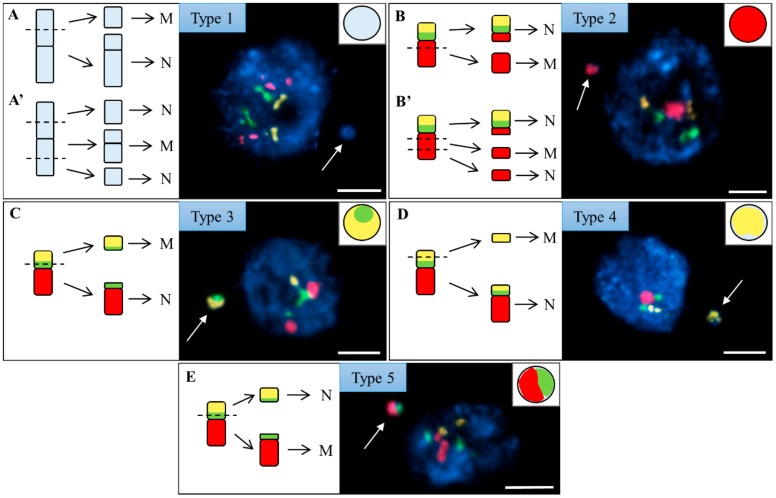
Brachypodium interphase nuclei with micronuclei induced by MH treatment and X-radiation, mcFISHed with the short (green) and long arm (red) Bd5-specific BAC probes and a 25S rDNA probe (yellow). The chromatin was stained with DAPI (blue), and white arrows indicate the micronuclei. Various types of micronuclei are distinguished and their detailed composition is described in the main text. The diagrams next to the photomicrographs show the putative origins of the micronuclei from the (**A**–**E**) distal or (**A’**,**B’**) interstitial chromosome regions. N, nuclei. MN, micronuclei. Transverse dashed lines indicate the DSBs. Scale bar = 5 µm.

Micronuclei were classified into five different types (Figure 6). The FISH-negative type 1 micronuclei probably originated from the distal (Figure 6A) or interstitial (Figure 6A’) fragments of unknown chromosomal origin. Type 2 was characterised by the presence of one signal derived from the Bd5 long arm. Depending on the number of DSBs, there are two theoretical possibilities of its origin—either from a fragment of the long arm of Bd5 that contains the telomere (Figure 6B) or from the interstitial fragment of this chromosome arm (Figure 6B’). The presence of one 25S rDNA signal and one BAC signal of the short arm defined the composition of micronucleus type 3 (Figure 6C). Because only one copy of the 25S rDNA signal is present in the original nucleus, the assumption is that a single DSB occurred within the chromatin region covered by the short arm Bd5-specific BAC pool. Micronucleus type 4 was composed exclusively of one 25S rDNA signal (Figure 6D). Because two yellow signals are observed in the nucleus, it seems likely that this micronucleus type resulted from a single DSB within the 35 rDNA sequences. Micronucleus type 5 contains two signals—one related to the long arm Bd5-specific BAC pool and the other derived from the short arm-painting Bd5 probe (Figure 6E). Such a composition implies that it was formed from a large centric fragment of Bd5 consisting of the entire, long arm and a proximal fragment of the short arm.

**Figure 7:**
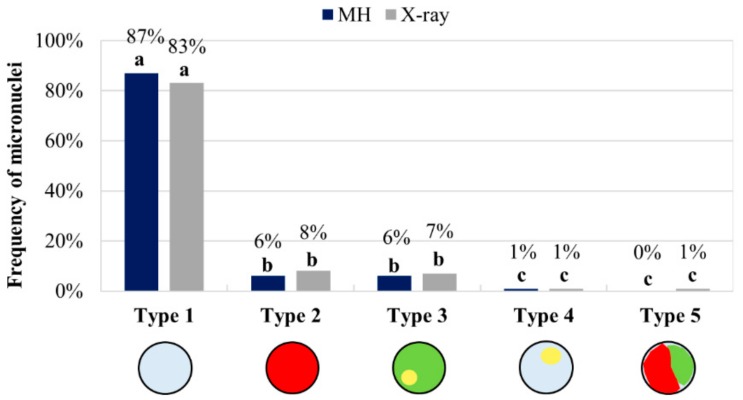
Frequencies of the five types of micronuclei following MH treatment and X-rays. The datasets for the two concentrations of MH and two doses of X-radiation were each combined. Means followed by the same letter (a, b, and c) are not significantly different from each other based on the parametric analysis of variance and post hoc LSD test (*p* < 0.05).

The relative frequencies of the different types of micronuclei are shown in Figure 7. The two datasets from two concentrations of MH and two doses of X-rays were combined as before, as they were not significantly different.

ANOVA and LSD multiple comparison tests once again showed significant differences (*p* < 0.05) in the frequencies of the various micronuclei types. As in the case of Bd4, the frequencies of the MH- and X-ray-induced micronuclei type 1 were shown to be significantly different from all of the other types (Table S4). No statistically significant differences were detected between the frequencies of the four other types.

FISH-negative micronuclei (type 1) had the highest frequency after both the MH treatment (87%) and X-radiation (83%). This was expected, as MH treatment and X-radiation are both strong inducers of micronuclei type 2, which are derived from a fragment of the long arm of Bd5 (6% and 8%, respectively). Surprisingly, type 3, 4, and 5 micronuclei derived from the short arm had a similar aggregate frequency after both MH treatment (7%) and X-radiation (9%). This demonstrates that DSBs are equally likely to occur in both arms of Bd5 despite the fact that the long arm is twice as long as the short arm [40]. However, type 3 micronuclei were the most abundant, suggesting that proximal chromosome regions of the short arm of Bd5 may be more prone to the effects of the mutagens.

In contrast, the secondary constriction/satellite of Bd5 with a 35S rDNA locus seems to be a “cold spot” of mutation, since DSBs rarely occur within this chromosome region after either MH treatment (1%) or X-radiation (1%). This observation is consistent with our previous findings [39]. However, in some plants 35S rDNA sequences tend to break and are designated fragile sites [41,42,43,44,45]. Fragile sites were originally defined in humans as regions that form non-staining gaps, constrictions, or breaks in one or both of the chromatids of metaphase chromosomes [46,47]. Within a plant genomic context 35S rDNA have been indicated as “hot spot”. Thus, it has been suggested that 35S rDNA is predisposed to anaphase-bridge cycles in telomerase-deficient *Arabidopsis thaliana* [48]. Moreover, an analysis of MH-treated barley cells showed that the duplication of a chromosome fragment included the 35S rDNA region [22]. On the other hand, a study of X-radiated *Lolium multiflorum* was in agreement with our results in suggesting that 35S rDNA was a “cold spot” of mutation. Eukaryotic rRNA genes are organised into numerous, tandemly repeated clusters, which may contribute up to 10% of a genome. In yeast, these numerous gene copies enable the repair of rDNA breakages through cohesion between sister chromatids [49,50]. A similar mechanism may occur in plant cells, resulting in the low frequency of DSBs in 35S rDNA we observed in Brachypodium cells subjected to MH treatment and X-radiation. Another reason why 35S rDNA seems to be a “cold spot” may be linked with our bodyguard hypothesis, which suggest a protective role of the constitutive heterochromatin for euchromatin from mutagens [5]. The loci that contain actively transcribed rRNA genes (known as nucleolar organising regions, NORs) may be protected by heterochromatic blocks which are localised around a secondary constriction and “absorb” the effects caused by mutagens.

## 3. Materials and Methods

### 3.1. Plant Material and Mutagenic Treatment

The experiments were performed with the *B. distachyon* (2*n* = 10) reference genotype Bd21. The seed material was obtained from the collection held by the United States Department of Agriculture’s National Plant Germplasm System. The seeds were treated with maleic hydrazide (MH; Sigma-Aldrich, St. Louis, MO, USA) at concentrations of 3 and 4 mM and X-radiation at doses of 125 and 150 Gy using a previously described procedure [25]. After the mutagenic treatment, the seeds were grown on filter paper moistened with tap water for three days at room temperature in the dark. Seedlings were collected and fixed in a 3:1 (*v*/*v*) mixture of 100% ethanol/glacial acetic acid at 4 °C overnight and then stored at −20 °C until they were used.

### 3.2. Root Meristem Preparation

Cytogenetic preparations were made according to the methodology of Jenkins and Hasterok [51]. Briefly, the fixative was removed by washing the excised roots in a 10 mM citric acid–sodium citrate buffer (pH 4.8) for 15 min. Enzymatic digestion was effected by a mixture consisting of 6% (*v*/*v*) pectinase (Sigma-Aldrich), 1% (*w*/*v*) cellulase (Sigma-Aldrich) and 1% (*w*/*v*) cellulase ‘Onozuka R-10′ (Serva, Heidelberg, Germany) for 1.5 h at 37 °C. Then, the meristems were dissected from the root tips and dispersed in a drop of 45% acetic acid.

### 3.3. DNA Probes and FISH

The BAC clone arrays that were used as the painting probes were obtained from two genomic DNA libraries of Brachypodium, BD_ABa and BD_CBa, which were constructed and described earlier [52]. Two different combinations of BACs were used as probes in the experiments to paint chromosomes Bd4 and Bd5, respectively (Tables S1 and S2). The selected BACs were divided into pools of 6–10 clones each as described in Idziak et al. [53] and isolated together using a standard alkaline lysis method. The BAC DNA was labelled by nick translation with digoxigenin-11-dUTP (Roche, Basel, Switzerland) for the short chromosome arms and with tetramethylrhodamine-5-dUTP (Roche) for the long arms. A 2.3 kb *Cla*I fragment of the 25S rDNA gene of *A*. *thaliana* [54] and the clone pTa794, which contained the 5S rRNA gene from wheat [55], were labelled with biotin-16-dUTP (Roche) and used in two different FISH experiments.

FISH was performed following the procedure of Jenkins and Hasterok [51] with minor modifications. In brief, the hybridisation mixture consisted of 50% deionised formamide, 10% dextran sulphate, 2 × SSC, 0.5% SDS, and 2.5–3.0 ng/μL of labelled DNA. The mixture was pre-denatured for 10 min at 75 °C, applied to the slides and denatured together at 75 °C for 4.5 min before being incubated in a moist chamber overnight at 37 °C. After hybridisation, the slides were washed in 20% formamide in 2 × SSC (10 min, 37 °C), which is equivalent to a ~59% stringency. The immunodetection of digoxigenated and biotinylated DNA probes was performed using FITC-conjugated anti-digoxigenin antibodies (Roche) and Alexa Fluor 647-conjugated anti-biotin antibodies (Jackson Immuno Research Laboratories, West Grove, PA, USA), respectively. The preparations were mounted and counterstained in Vectashield (Vector Laboratories, Peterborough, UK) that contained 2.5 μg/mL DAPI (Serva).

### 3.4. Image Acquisition and Processing

All photomicrographs were acquired using an AxioCam MRm (Zeiss, Oberkochen, Germany) high-sensitivity monochromatic camera attached to a wide-field Axio Imager.Z.2 (Zeiss) fluorescence microscope. All of the images were then digitally coloured using Wasabi (Hamamatsu Photonics, Bridgewater, NJ, USA), uniformly processed to improve contrast and brightness (if required) and then superimposed using Photoshop CS3 (Adobe, San Jose, CA, USA). In order to estimate the total frequency of the DAPI-stained micronuclei at least 2000 interphase nuclei were analysed for each experimental group. In the FISH experiments, the frequencies of micronuclei with specific signals and without signals were calculated. For each experimental group about 5500 nuclei, including 300 nuclei with micronuclei arising from one meristem, were analysed on three individual slides.

### 3.5. Statistical Analyses

The normal distribution of the frequencies of nuclei with micronuclei was assumed [25]. Possible differences between the frequencies of micronuclei after either the MH treatment or X-radiation were verified using the parametric ANOVA test preceded by the Levene’s test for equality of variances. A post hoc comparison of the means was performed using the Least Significant Difference (LSD) test.

## 4. Conclusions

We have used BAC-FISH-based chromosome painting to explore in detail the composition, origin, and mechanisms of micronuclei formation in Brachypodium. We have deduced that DSBs induced by MH treatment and X-radiation are site-specific in chromosome Bd4. Discrimination between different regions of chromosome Bd5 allowed us to conclude that the proximal region of its short arm is more prone to mutations. Furthermore, we showed conclusively that 5S rDNA and 35S rDNA do not preferentially break after MH treatment and X-radiation. Our findings demonstrate the potential of Brachypodium as a useful model plant in mutagenesis studies.

## Supplementary Materials

Supplementary materials can be found at https://www.mdpi.com/1422-0067/20/11/2848/s1. Table S1: Characteristics of bacterial artificial chromosome (BAC) clones used for the specific painting of *Brachypodium distachyon* chromosome Bd4, Table S2: Characteristics of bacterial artificial chromosome (BAC) clones used for the specific painting of *Brachypodium distachyon* chromosome Bd5, Table S3: Detailed statistical analyses related to the data presented in Figure 3. Values are means±standard error (*n* = 3). Means followed by the same letter (a, b, c) are not significantly different from each other basing on the parametric analysis of variance and post hoc LSD test (*p* < 0.05), Table S4: Detailed statistical analyses related to the data presented in Figure 6. Values are means±standard error (*n* = 3). Means followed by the same letter (a, b, c) are not significantly different from each other basing on the parametric analysis of variance and post hoc LSD test (*p* < 0.05).
