# Supplementary material for: Detecting Brachypodium distachyon Chromosomes Bd4 and Bd5 in MH- and X-Ray-Induced Micronuclei Using mcFISH
Source: Int J Mol Sci. 2019 Jun 11;20(11):2848. doi: 10.3390/ijms20112848 (PMC6612364; doi:10.3390/ijms20112848)
Supplement: Supplementary file 1 [file ijms-20-02848-s001.ZIP › ijms-490628-Table S1.pdf]

TABLE S1. Characteristics of bacterial artificial chromosome (BAC) clones used for the specific painting of *Brachypodium distachyon* chromosome Bd4.

Short (S) arm

Clone name	Start (bp)	End (bp)	Repeat content (%)
b0022F16	1375208	1513070	17.05
a0029D03	1509327	1527857	14.80
b0011J07	1877049	2006504	13.50
b0030B12	2007584	2157984	7.19
a0008M09	3005577	3057490	10.02
b0031P08	4003479	4005284	0.00
b0020L19	4509871	4652127	17.87
b0015K04	5000667	5192928	12.52
b0015K04	5192351	5334608	20.17
a0021K11	5356098	5506730	12.85
b0040J03	7830905	8001843	19.13
b0021B09	9502901	9667864	12.35
a0039N16	9858184	10002657	14.32
a0043D11	11006774	11150531	22.28
a0038M23	13356537	13506604	23.57
a0004L13	13506625	13718798	11.46
a0010I18	14002249	14164264	10.04
a0028M13	14467770	14501426	10.11
a0006A19	15500025	15633790	10.64
b0023J07	18378813	18509732	28.56

Long (L) arm

Clone name	Start (bp)	End (bp)	Repeat content (%)
a0047K04	27639991	27795427	18.44
b0012C05	28999425	29071930	12.74

Clone name	Start (bp)	End (bp)	Repeat content (%)
a0006J17	29358544	29516826	9.42
b0027J13	30855184	31004161	28.40
a0013N14	31000455	31132079	9.47
a0020D08	32504625	32642850	8.94
b0033J04	32835278	33001029	24.02
a0029H13	33008088	33024284	20.44
b0005K02	33865813	34006488	20.04
b0002F10	34027870	34141505	10.19
a0021F10	35000545	35176627	26.57
a0011F18	36857616	37009036	26.55
a0031C04	37506966	37653676	24.29
a0004J07	38767257	38918272	6.73
b0021H03	38925520	39066237	14.28
b0035E05	39350118	39526113	6.40
b0014B09	39506174	39642910	17.79
a0024O22	39853958	40006315	16.05
b0041J05	40005710	40200691	17.39
a0032J21	40499878	40546546	5.53
a0003H15	40835257	41003446	12.86
b0047A04	41006415	41014781	22.45
a0047P14	41364258	41502074	13.90
a0026J09	41502088	41633709	24.33
b0032E02	41852352	42005820	21.66
b0026E20	42346424	42505268	15.02
b0038H23	42789149	43003220	6.17
b0023G20	43352742	43500050	9.76
b0019I11	43876622	44010479	6.16
a0004A24	44343043	44510220	7.93
a0043N14	45504848	45661980	6.49
b0043F05	46001501	46132046	15.43
a0030C04	46357221	46502222	7.16

Clone name	Start (bp)	End (bp)	Repeat content (%)
a0004O19	46502230	46658753	9.01
b0031I03	46846042	47008130	20.21
b0026N15	47003712	47134381	8.51
a0017H17	47506772	47643218	6.97
a0024E12	48004859	48154124	3.07
a0041I03	48350055	48507632	9.05
