# Supplementary material for: Detecting Brachypodium distachyon Chromosomes Bd4 and Bd5 in MH- and X-Ray-Induced Micronuclei Using mcFISH
Source: Int J Mol Sci. 2019 Jun 11;20(11):2848. doi: 10.3390/ijms20112848 (PMC6612364; doi:10.3390/ijms20112848)
Supplement: Supplementary file 1 [file ijms-20-02848-s001.ZIP › ijms-490628-Table S2.pdf]

TABLE S2. Characteristics of bacterial artificial chromosome (BAC) clones used for the specific painting of *Brachypodium distachyon* chromosome Bd5.

Short (S) arm

Clone name	Start (bp)	End (bp)	Repeat content (%)
a0018K07	853205	1009526	16.20
a0019O20	1091367	1236179	16.00
b0033P01	2501695	2512065	1.35
a0009O09	2504345	2648229	16.66

Long (L) arm

Clone name	Start (bp)	End (bp)	Repeat content (%)
a0045F23	13499779	13653343	18.89
b0030K21	15304885	15505515	22.56
a0026M04	17499731	17633162	23.72
a0023L21	17634500	17679830	4.89
a0001F13	17802975	18003203	23.52
a0017D24	18003221	18155770	16.96
b0042J14	18312982	18503857	19.62
a0046O09	20358624	20503060	24.99
b0024J19	20845837	21003148	11.02
b0033K07	21003184	21110356	11.50
b0037B05	21507488	21710347	23.77
b0016H11	21877774	22006306	16.69
b0041K21	23480858	23500119	1.39
b0032J06	23870997	24003288	6.19
a0045J11	24003128	24154997	9.30
a0026B16	24841254	25002060	5.96

Clone name	Start (bp)	End (bp)	Repeat content (%)
a0031B15	25503136	25695070	4.94
a0023B07	25746075	25906029	8.99
a0019J13	25906054	26098440	3.35
