# Supplementary material for: Detecting Brachypodium distachyon Chromosomes Bd4 and Bd5 in MH- and X-Ray-Induced Micronuclei Using mcFISH
Source: Int J Mol Sci. 2019 Jun 11;20(11):2848. doi: 10.3390/ijms20112848 (PMC6612364; doi:10.3390/ijms20112848)
Supplement: Supplementary file 1 [file ijms-20-02848-s001.ZIP › ijms-490628-Table S3.pdf]

TABLE S3. Detailed statistical analyses related to the data presented in Figure 3. Values are means±standard error ($n = 3$). Means followed by the same letter (a, b, c) are not significantly different from each other basing on the parametric analysis of variance and *post hoc* LSD test ($p < 0.05$).

MH Type 1	84.00±1.00 a
MH Type 2	4.00±0.58 b
MH Type 3	5.00±0.58 b
MH Type 4	5.00±1.15 b
MH Type 5	1.00±0.58 c

X-ray Type 1	84.00±1.45 a
X-ray Type 2	6.00±1.15 b
X-ray Type 3	4.00±0.58 b
X-ray Type 4	6.00±0.58 b
X-ray Type 5	0.00±0.00 c
