# Supplementary material for: Detecting Brachypodium distachyon Chromosomes Bd4 and Bd5 in MH- and X-Ray-Induced Micronuclei Using mcFISH
Source: Int J Mol Sci. 2019 Jun 11;20(11):2848. doi: 10.3390/ijms20112848 (PMC6612364; doi:10.3390/ijms20112848)
Supplement: Supplementary file 1 [file ijms-20-02848-s001.ZIP › ijms-490628-Table S4.pdf]

TABLE S4. Detailed statistical analyses related to the data presented in Figure 6. Values are means±standard error ($n = 3$). Means followed by the same letter (a, b, c) are not significantly different from each other basing on the parametric analysis of variance and *post hoc* LSD test ($p < 0.05$).

MH Type 1	86.00±1.73 a
MH Type 2	6.00±0.58 b
MH Type 3	6.00±1.00 b
MH Type 4	1.00±0.58 c
MH Type 5	0.33±0.33 c

X-ray Type 1	83.00±1.53 a
X-ray Type 2	8.00±1.00 b
X-ray Type 3	7.00±1.15 b
X-ray Type 4	1.00±0.58 c
X-ray Type 5	1.00±0.00 c
